# Setting Behavior and Phase Evolution on Heat Treatment of Metakaolin-Based Geopolymers Containing Calcium Hydroxide

**DOI:** 10.3390/ma15010194

**Published:** 2021-12-28

**Authors:** Byoungkwan Kim, Sujeong Lee, Chul-Min Chon, Shinhu Cho

**Affiliations:** 1 Division of Advanced Nuclear Engineering, Pohang University of Science and Technology, Pohang 37673, Korea; kwan928@postech.ac.kr; 2 Mineral Resources Research Division, Korea Institute of Geoscience and Mineral Resources, Daejeon 34132, Korea; 3 Department of Resources Recycling Engineering, University of Science and Technology, Daejeon 34113, Korea; 4 Geological Environment Division, Korea Institute of Geoscience and Mineral Resources, Daejeon 34132, Korea; femini@kigam.re.kr; 5 Advanced Materials Research Team, Hyundai Motor Group, Uiwang 16082, Korea; s.cho@hyundai.com

**Keywords:** fast setting, calcium hydroxide, C-S-H, metakaolin, geopolymer

## Abstract

The setting behavior of geopolymers is affected by the type of source materials, alkali activators, mix formulations, and curing conditions. Calcium hydroxide is known to be an effective additive to shorten the setting period of geopolymers. However, there is still room for improvement in the understanding of the effect of calcium hydroxide on the setting and phase evolution of geopolymers. In this study, the setting behavior and phase evolution of geopolymer containing calcium hydroxide were investigated by XRD analysis. The setting time of the geopolymer was inconsistently shortened as the amount of calcium hydroxide increased. A low calcium hydroxide dose of up to 2% of the total mix weight could contribute to the enhancement of compressive strength of geopolymers besides a fast-setting effect. The C-S-H gel is rapidly precipitated at the early stage of reaction in geopolymers containing high calcium hydroxide with some of the calcium hydroxide remaining intact. The ex-situ high-temperature XRD analysis and Rietveld refinement results revealed that geopolymer and C-S-H gel transformed into Si-rich nepheline and wollastonite, respectively. The wollastonite was also observed in heat-treated geopolymers with a low calcium hydroxide dose. It is believed that C-S-H gel can be precipitated along with geopolymers regardless of how much calcium hydroxide is added.

## 1. Introduction

Geopolymers are inorganic binder materials synthesized by combining amorphous aluminosilicate materials such as fly ash and metakaolin with alkaline solutions between ambient and low temperatures below 100 °C [1,2]. As compared to ordinary Portland cement (OPC) which is hardened via hydration reaction, the hardening of geopolymers is caused by polycondensations between aluminate and silicate species which are released from source materials and alkaline solution [1]. Randomly connected Si and Al tetrahedrons are linked by sharing oxygen anions form the network structure of geopolymers. The negative charge of structure created by Si substitution of Al is compensated by alkali metal ions such as Na and K [1]. Geopolymers exhibit outstanding performances which are hardly obtained in OPC such as high early-age strength, good chemical stability, thermal resistance, and immobilization of toxic elements [3,4,5,6,7,8].

Regardless of the source materials, it takes a longer period of time for geopolymers to set compared to OPC, which takes about 10 h for the final setting at ambient temperature [9]. The initial setting of fly ash-based geopolymer using low calcium fly ash (class F) takes more than a day at ambient temperature [10]. In the case of metakaolin-based geopolymer, the final setting time is about 20 h at room temperature, which is faster than fly ash-based geopolymer [11,12,13]. This is because the combination of several factors such as mix formulation, including a targeted Si/Al molar ratio in the mix, curing temperature, the reactivity of source material, and the pH of an alkaline activator affects the setting of geopolymers. If it is possible to reduce or control the setting time of geopolymers in the desired time, they could be used in a wider variety of fields where OPC and rapid-setting cement are used, such as in 3D printing and building maintenance. In order to control the setting of geopolymers, three setting control methods have been suggested: (1) the increasing of curing temperatures (heat curing), (2) the use of source materials with a high calcium content, and (3) the addition of calcium compounds [14,15,16]. Heat curing can significantly reduce setting time and accelerate geopolymerization [15], but it is difficult to apply to the production of site-poured concrete. The use of source material with high calcium content such as class C fly ash can lead to fast setting at room temperature. The initial and final setting times for fly ash-based geopolymers fabricated using class C fly ash and cured at room temperature are 30 min and 1 h, respectively [16]. However, it is difficult to quantitatively control the setting of geopolymers because setting time is only unaffected by the calcium content in the fresh mix. In addition, the bad workability of a mixture which is caused by fast setting needs to be improved for practical use [5].

The addition of calcium compounds such as calcium oxide, calcium hydroxide, and calcium rich by-product such as ground granulated blast furnace slag can also reduce the setting time of geopolymers [12,17,18,19,20,21,22]. The alkali activated materials using calcium rich by-products form two-dimensional structure [23,24]. It may affect the properties of geopolymer such as long-term properties and resistance to the chemical agents, but its effect on the geopolymer structure is unclear [23,24]. Considering the disadvantages of heat curing and the use of high calcium source materials, the addition of calcium compound is more beneficial since the appropriate amount of calcium compound is effective for fast setting as well as the enhancement of compressive strength [17,19,20,21]. The most effective calcium source is calcium hydroxide, though more understanding of the setting behavior and phase evolution of geopolymers containing calcium still needs to be achieved to fully understand its mechanisms [12,17,18,19,20,21,22].

The objective of this study is an investigation of the setting behavior and phase evolution of geopolymers containing calcium hydroxide. The appropriate amount of calcium hydroxide was added to the geopolymer which is fabricated using pure aluminosilicate material such as metakaolin. The stoichiometry of metakaolin-based geopolymers was designed with NaSi_2_AlO_6_·5H_2_O and calcium hydroxide was added as 2%, 4%, 8%, and 16% by weight of the geopolymer mixture. The variations of setting behavior, compressive strength, and the phase evolution of geopolymers depending on the calcium hydroxide content were investigated. In addition, the Rietveld refinement based on X-ray diffraction was performed to indirectly understand the role of calcium in geopolymer structures and the high-temperature phase of Si-rich geopolymer after ex-situ heat treatment. The low-carnegieite and Si-rich nepheline were newly refined to understand the phase transition of geopolymer containing high Si content. The scientific findings of this study will contribute to the understanding of the setting behavior and phase evolution of geopolymers containing calcium hydroxide.

## 2. Materials and Methods

### 2.1. Characterization of Metakaolin

Geopolymers were synthesized from commercial metakaolin (MetaMax^®^, BASF, Ludwigshafen, Germany). The chemical composition of metakaolin was analyzed using X-ray fluorescence spectroscopy (Shimadzu Sequential XRF-1800, Shimadzu, Japan). Quantitative X-ray diffraction phase analysis was conducted employing the DIFFRAC.EVA V4.2 (Bruker-AXS, Karlsruhe, Germany), PDF-2 release 2016 (ICDD, Newtown Square, PA, USA), and TOPAS 5 software (Bruker-AXS). Calcium fluoride (CaF_2_, 99.985%, Alfa aesar, Ward Hill, MA, USA) was used as an internal standard to comprise 10.0000% of the sample weight. The mixture of metakaolin and calcium fluoride was ground in a micronizer mill (McCrone, Westmont, IL, USA) for 5 min. X-ray diffraction patterns were obtained using a D8 Advance diffractometer (Bruker-AXS, Karlsruhe, Germany) over a 2θ range from 5 to 65 with a step size 0.01° or 1 s/step. To evaluate the instrument-derived errors, an X-ray diffraction pattern of lanthanum hexaboride (LaB_6_, SRM 660b, NIST, Gaithersburg, MD, USA) was also obtained employing the same diffraction conditions.

### 2.2. Synthesis of Metakaolin-Based Geopolymer

Metakaolin-based geopolymers were synthesized by mixing the metakaolin and alkaline activators to achieve the molar ratio of Na_2_O:Al_2_O_3_:SiO_2_:H_2_O = 1:1:4:10 in geopolymers (Table 1). Fumed silica (EH-5, Cabot corporation, Boston, MA, USA assay 99.9%), sodium hydroxide (NaOH, Daejung chemical, Siheung-si, Korea, assay 97.0%), and distilled water were mixed at 25 °C for ≥20 h before use and then added to metakaolin. Calcium hydroxide (Ca(OH)_2_, Kyoto, Japan, Yakuri chemical, assay 96.0%) was added from 2% to 16% of the total mix weight just before the end of the geopolymer mixing process.

### 2.3. Measurement of Setting Time

Geopolymers for measuring the final setting time were synthesized by using a planetary mixer (ARE-310, Thinky, Tokyo, Japan). Metakaolin and alkaline activator were mixed for 4 min at 1400 RPM, and then defoamed for 3 min at 1700 RPM. The geopolymer mixture was poured into a 200 mL PP beaker to fill a height of 30 mm of the beaker subsequent to mixing. Setting time was measured in accordance with ASTM C191 (Standard Test Methods for Time of Setting of Hydraulic Cement by Vicat Needle) utilizing a Vicat needle apparatus.

### 2.4. Measurement of Compressive Strength

Geopolymers for measuring the 7-d compressive strength were prepared using a Kenwood mixer (Kenwood kitchen appliances, Hertfordshire, UK). Metakaolin and alkaline activator were mixed for 2 min at low speed, and then mixed for 5 min at high speed. The geopolymer mixtures were filled into cube molds (5 cm × 5 cm × 5 cm) according to ASTM C109 (Standard Test Method for Compressive Strength of Hydraulic Cement Mortars). One-third of the mold volume was filled and then tapped to remove air bubbles trapped during the mixing process. The molded geopolymers were cured at room temperature for 7 days after sealing to prevent the evaporation of moisture. The 7-d compressive strength of geopolymers was measured with MTS 815 rock mechanics test machine (MTS system corporation, Eden Prairie, MN, USA) at a loading rate of 5.5 × 10^−3^ mm/s.

### 2.5. X-ray Diffraction Analysis of Geopolymers

X-ray diffraction analysis for hardened geopolymers and heat-treated geopolymers was performed using a D8 Advance diffractometer. Diffraction patterns of hardened geopolymers were obtained over a range of 8–80° 2θ with a step size of 0.02°, and a scan speed of 0.2 s/step. For the ex-situ high-temperature X-ray diffraction analysis, geopolymer fragments were obtained from a fresh fracture of hardened geopolymer. The samples were crushed in agate mortar with the geopolymer powder then sieved to a size of −150 + 106 μm. After placing about 5 g of sieved geopolymer powder in a nickel crucible, it was heated at 900 °C for 30 min at a heating rate of 5 °C/min using an electric furnace. The qualitative analysis of obtained X-ray diffraction patterns was conducted utilizing DIFFRAC.EVA V4.2 and PDF-2 Release 2016. Structure refinement employing the Rietveld of newly formed phases was performed using TOPAS 5 software.

## 3. Results and Discussion

### 3.1. Characteristics of Metakaolin

**Figure 1:**
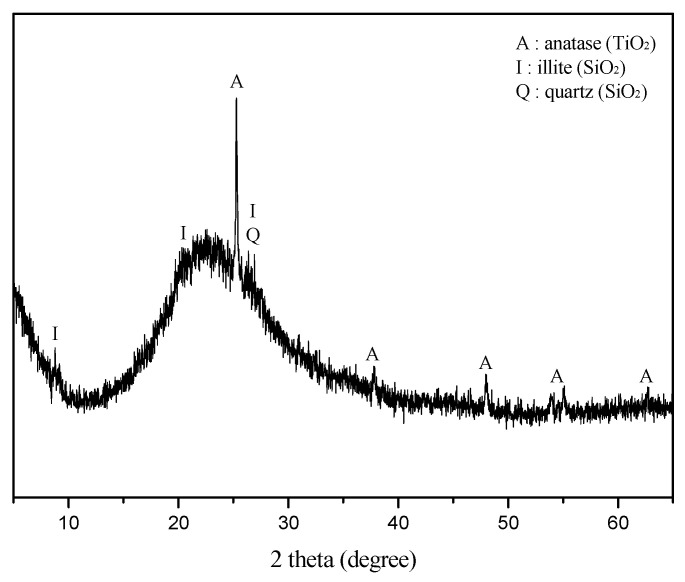
X-ray diffraction pattern of metakaolin in this study. Metakaolin was composed of amorphous and several crystalline phases such as anatase, illite, and quartz as impurities. The center of the amorphous hump was located at 2θ 22.9°.

The main components of metakaolin were SiO_2_ and Al_2_O_3_, although it contained roughly 1.5 wt% of TiO_2_ as a major impurity which is identified as anatase in XRD analysis (Table 2 and Figure 1). Metakaolin was mainly composed of amorphous materials while containing crystalline impurities such as illite, quartz, and anatase (Figure 1). The center of amorphous diffraction hump of metakaolin was located at roughly 2θ 23° (Figure 1). The mix formulation of geopolymers was obtained by considering both bulk Si and Al contents in Table 2 as all reactive because the trace amount of crystalline SiO_2_ in illite and quartz could be ignored.

### 3.2. Setting Behavior and Compressive Strength of Metakaolin-Based Geopolymers Containing Calcium Hydroxide

**Figure 2:**
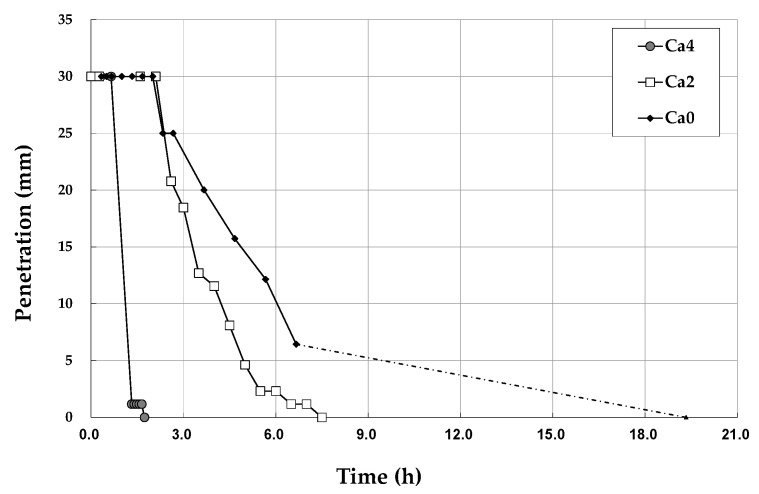
Setting behavior of metakaolin-based geopolymers. The addition of 4% calcium hydroxide promoted a fast set by interlocking coagulate products from the outset.

The final setting time of Ca0 was not able to be continuously measured due to the essential limitations of the Vicat needle test, which is performed manually. Although the exact final setting time of pure geopolymer cannot be determined here, it can be concluded that the hardening of the geopolymer having the targeted chemical composition was accomplished within 19 h post mixing as proved in previous studies (Figure 2). The setting time substantially decreased by adding calcium hydroxide. There was, however, no significant difference in the initial setting time between 2.3 h for Ca0 and 2.1 h for Ca2. Then, Ca2 showed a faster setting duration than Ca0 after 2.6 h (Figure 2). When the content of calcium was doubled in Ca4, the initial setting time was dramatically reduced to 1.3 h (Figure 2). It took just 1.7 h to reach the final setting in Ca4 (Figure 2). Geopolymers containing high amounts of calcium hydroxide (Ca8 and Ca16) were too rapidly hardened even during mixing to measure the setting time.

The addition of calcium hydroxide contributed to the superior mechanical property of calcium-containing geopolymers. The compressive strength of Ca2 was 76 MPa, which was higher than 68 MPa of the Ca0 (Table 3). In other words, the addition of 2% calcium hydroxide appears less effective in decreasing the initial setting time while beneficial for a fast final set. By contrast, 4% calcium hydroxide inclusion promoted a fast set by interlocking coagulate products from the outset. The polycondensation of geopolymer gel occurs slowly [11,12,13] and the setting time of pure geopolymers is mainly controlled by the alumina content, because aluminate is more readily soluble in metakaolin [25]. Condensation between silicate entities occurs slower than aluminate-silicate condensation so the setting time increases with the increasing SiO_2_/Al_2_O_3_ ratio of the initial mixture [25]. According to the dynamic rheological measurement study during geopolymerization [26], the size of alkali cations affects the dissolution and condensation rates of aluminate and silicate entities. The role of calcium in fast set geopolymers should be deliberately investigated due to the fact that sodium is fully added to compensate the negative charge in the formulation (Table 1) and coexists with extra cation and calcium in the mixture.

The Ca4 produced by the Kenwood mixer hardened too quickly to be molded for strength measurement. It set faster than the one synthesized using the plenary mixer which is known to be much more effective in geopolymer mixing. A high-shear mixer such as the plenary mixer contributes to obtain high strength geopolymers to ensure full reactivity [27]. Aluminate and silicate species probably dissolved more slowly in the mixture than one produced by the plenary mixer for the Vicat test. As a result, polycondensation of entities may be retarded but nevertheless contain high calcium content which possibly played a crucial role in decreasing the setting time in Ca4. The calcium hydroxide promotes the dissolution of metakaolin in geopolymer activated by alkali silicate solution [12]. The 5- and 6-coordinated Al present in the metakaolin convert to 4-coordinated Al during the geopolymerization [28]. In geopolymers containing calcium hydroxide, 5- and 6-coordinated Al is rapidly converted into 4-coordinated Al in the beginning of the geopolymerization. The percentage of 4-coordinated Al is higher than that of the geopolymer without calcium by at least 10% [11]. Thus, calcium hydroxide contributes to the fast dissolution of metakaolin and the promotion of geopolymer gel formation resulting in the fast setting of the geopolymer [11]. However, what is finally formed apart from geopolymers in extra alkali cations-containing geopolymers should be thoroughly investigated.

The number of network modifiers in the geopolymer structure may increase due to the addition of calcium hydroxide if calcium plays a role of network modifier. It may result in an increase in the number of non-bridging oxygens in the geopolymer network locally and may lead to depolymerization of geopolymers. Calcium can play different structural roles such as network modifier or charge compensator in a CaO-Na_2_O-Al_2_O_3_-SiO_2_ glass system, but it has an affinity for network modifiers [29,30]. It is known that network modifiers reduce the number of strong bonds in the glass and lower the melting point. The deteriorated consistency of calcium-containing Ca2 and Ca4 reflects that calcium did not contribute to the depolymerization. On the contrary, C-S-H gel can be precipitated easily if there is only a small amount of calcium in the silicate solution because the solubility product of C-S-H gel which can be formed by the interaction of calcium and silicate has a low value of 5.5 × 10^−49^ at room temperature [31,32]. Therefore, the increase in compressive strength can be attributed to the fast dissolution of metakaolin, the rapid formation of the geopolymer, and the precipitation of C-S-H gel concurrently. Although the microstructure of C-S-H gel was not confirmed in this study, its characteristic needle-like or reticular structure of C-S-H phases possibly played a part in the improvement of compressive strength of Ca2 compared with Ca0.

### 3.3. X-ray Diffraction Analysis of Metakaolin-Based Geopolymer Containing Calcium Hydroxide

**Figure 3:**
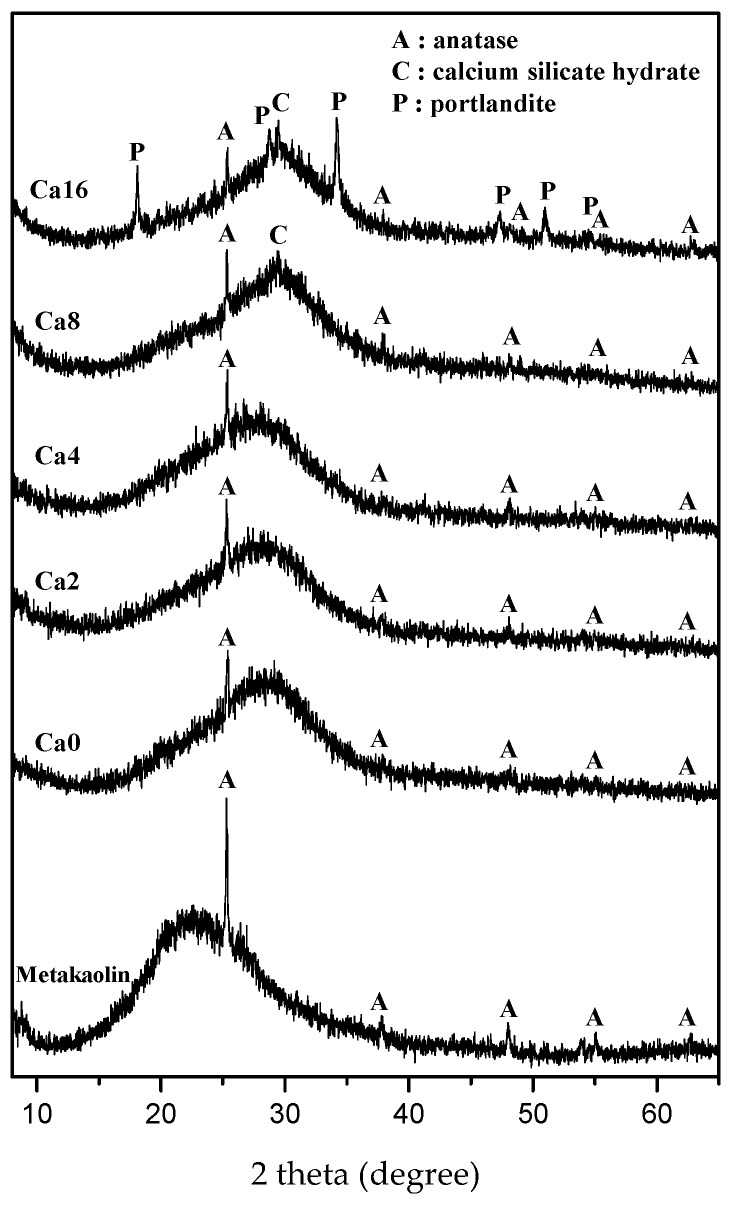
XRD patterns of pure geopolymer and geopolymers containing calcium hydroxide. The newly formed C-S-H gel and unreacted calcium hydroxide were observed in Ca8 and Ca16.

The XRD patterns of Ca0, Ca2, and Ca4 showed the broadening amorphous hump which indicates the geopolymer structure (Figure 3) [33]. On the other hand, a new cuspidal diffraction peak was recorded at about 2θ 29° in Ca8 and Ca16 which is higher 2θ compared with the amorphous hump of Ca0 (Figure 3). This cuspidal reflection at about 2θ 29° represents the C-S-H gel [34]. The crystalline calcium hydroxide, portlandite, was recognized as well in Ca16. (Figure 3). It is due to the common ion effect of calcium hydroxide and high pH environment in the alkaline activator. These conditions lead to a decrease in solubility of calcium hydroxide and result in the reduction of C-S-H gel precipitation [35,36,37]. In addition, the solubility of calcium hydroxide decreases with increasing temperature [38]. Geopolymerization is an exothermic reaction and releases the highest heat at the early stage of the reaction in which metakaolin is dissolved in the alkaline activator [39]. The exothermic heat released during dissolution of metakaolin can increase as calcium hydroxide content increases, as calcium hydroxide contributes to the fast dissolution of metakaolin [12,37]. As a result, the solubility of calcium hydroxide probably decreased in the geopolymer containing higher calcium hydroxide, with Ca16 here remaining intact.

**Figure 4:**
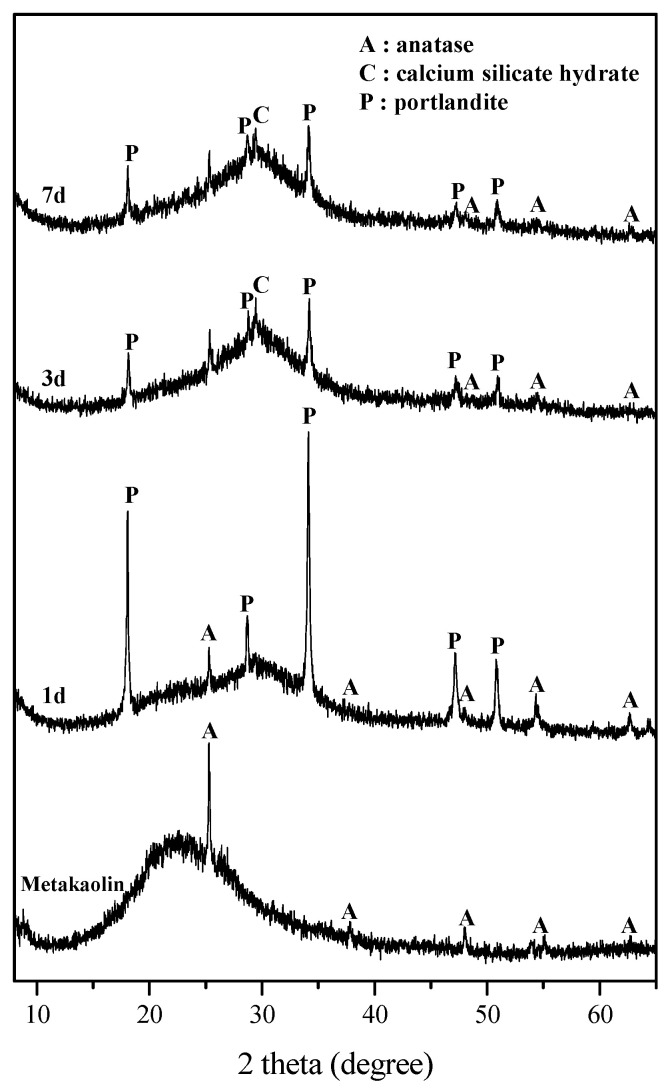
XRD patterns of Ca16 cured for one, three, and seven days. C-S-H gel is rapidly precipitated at the early stage of reaction and some of the calcium hydroxide remains intact.

The XRD analysis results of Ca16 showed C-S-H gel is rapidly precipitated at the early stage of reaction and some of the calcium hydroxide remains inert in hardened geopolymers (Figure 4). The Ca16 at the first day of curing was composed of geopolymer presenting an amorphous hump at 2θ 23°, with C-S-H gel presenting another hump at 2θ 23°, unreactive metakaolin, and portlandite (Figure 4). At three and seven days of curing, the shape of the amorphous humps of geopolymer and C-S-H gel became more symmetrical, and the intensity of portlandite peaks decreased (Figure 4). This showed that the dissolution of metakaolin and calcium hydroxide continued with the increase in the curing period (Figure 4). The continued dissolution of calcium hydroxide may supply calcium to the C-S-H gel with a low Ca/Si ratio formed at 1 day of curing, so that the C-S-H gel at a longer curing period probably has a higher Ca/Si ratio and the atomic ordering will be increased. Meanwhile, the XRD patterns of three and seven days of curing showed little difference and the intensities of undissolved calcium hydroxide were also similar (Figure 4). Therefore, it can be concluded that C-S-H gel is rapidly precipitated at the early stage of reaction and some of the calcium hydroxide remains inert due to the common ion effect, high pH environment, and exothermic heat of geopolymerization when added as an excessive amount to the geopolymer mixture.

### 3.4. Ex-Situ High-Temperature X-ray Diffraction Analysis of Metakaolin-Based Geopolymers Containing Calcium Hydroxide

**Figure 5:**
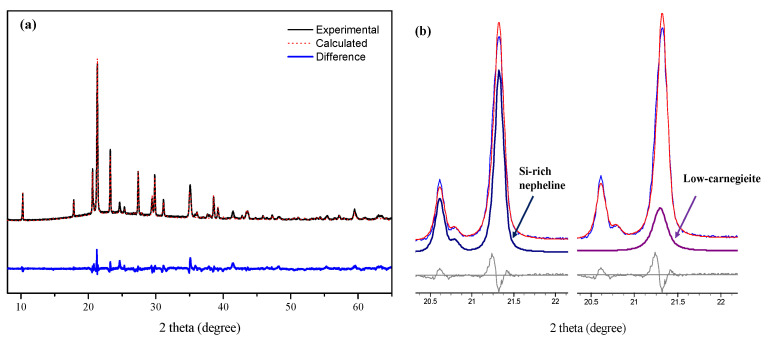
Rietveld refinement result of pure geopolymer heat treated at 900 °C (**a**); the crystal information of Si-rich nepheline and low-carnegieite was refined. The overlapped diffraction pattern of Si-rich nepheline and low-carnegieite was confirmed at 2θ 21.2° (**b**).

The crystalline phases of heat-treated metakaolin-based geopolymer varies with its chemical formulation and the cation of alkaline activator [40]. The geopolymers using potassium hydroxide as an alkaline activator transform into kalsilite (KAlSiO_4_) and leucite (KAlSi_2_O_6_) depending on the Si/Al ratio at temperatures above 1000 °C [40]. In the case of sodium hydroxide being used as an alkaline activator, geopolymers transform into nepheline (NaAlSiO_4_), jadeite (NaAlSi_2_O_6_), and albite (NaAlSi_3_O_8_) [41,42,43] depending on the Si content at temperatures above 800 °C. The high-temperature crystalline phase can be assumed to be jadeite because the Ca0 is Na-based and its Si/Al molar ratio was two. Jadeite is involved in a high-pressure phase and exhibits strong diffraction peaks at 2θ 30° and 31°. This is inconsistent with the strong diffraction peak at 2θ 23°of the newly formed phase. Rietveld refinement of XRD pattern presented that Si-rich nepheline and low-carnegieite were formed in heat-treated Ca0 geopolymer at 900 °C (Figure 5).

As mentioned earlier, the Si/Al molar ratio of nepheline is 1.0, yet several studies reported that nepheline was formed as a high-temperature crystalline phase even in geopolymers with a Si/Al molar ratio of 1.0 or higher [44,45]. Metakaolin-based geopolymers demonstrating a Si/Al molar ratio between 1.15 and 2.15 were crystallized to nepheline (PDF #00-035-0424) after heating at 900 °C, and the diffraction intensities of nepheline decreased as the Si/Al molar ratio of the geopolymer increased [44]. Another study reported on the formation of nepheline when the metakaolin-based geopolymers with a Si/Al molar ratio of 1.8 were heated at 1000 °C [44]. Nevertheless, the Si/Al molar ratio of geopolymers in these studies (Si/Al molar ratio = 1.15≥) [44,45] was higher than that of nepheline (Si/Al molar ratio = 1). The intensity ratio of the obtained peaks of this phase were also inconsistent with that of nepheline reported in [44,45]. These studies showed that nepheline could be formed via heat-treatment of geopolymers with a Si/Al ratio of more than 1. In conclusion, the high-temperature crystalline phase of geopolymers in this study is estimated as a Si-rich nepheline from the previous research results as well as the refinement analysis in this study.

**Figure 6:**
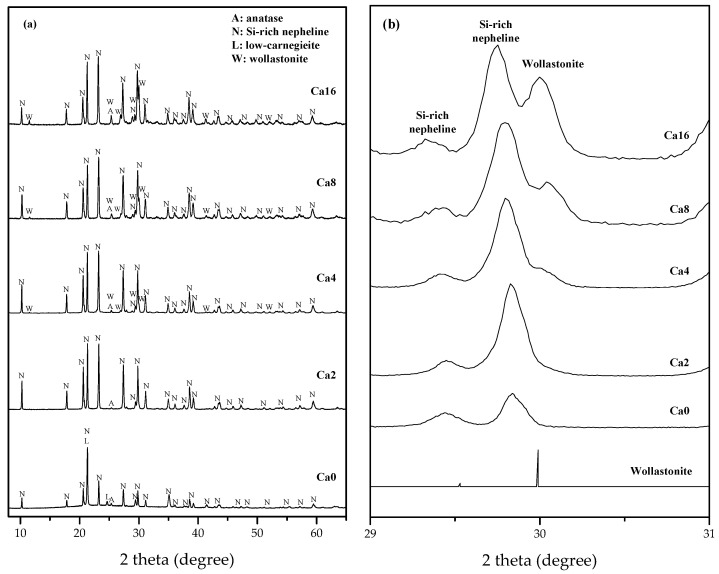
XRD analysis results of pure geopolymer and geopolymers containing calcium hydroxides post heat treatment (**a**) and 2θ range from 29° to 31° (**b**); Si-rich nepheline and low-carnegieite were observed in Ca0 geopolymers as high temperature crystalline phases. The low reflection of wollastonite was confirmed in Ca4.

The Ca0 showed the strongest XRD pattern at 2θ 21° while geopolymers containing calcium hydroxide represented it at 2θ 23° (Figure 6a). These differences may be due to the overlap of Si-rich nepheline and low-carnegieite diffraction patterns. Nepheline and low-carnegieite can be formed concurrently when the metakaolin-based geopolymer is heated at 900 °C, and the strongest diffraction peaks are overlapped at 2θ 21° [42]. Nepheline and carnegieite are polymorphisms with the same chemical composition, but with different crystal structures. The Si/Al molar ratio of geopolymers that were synthesized in this study was higher than low-carnegieite. In addition, it is known that low-carnegieite containing high Si content does not exist. According to a previous study, when metakaolin and Na_2_CO_3_ are milled and then heated at 600–800 °C, carnegieite can be formed [46]. Hence, the formation of low-carnegieite is presumably caused by the reaction between Na which is released from the alkaline activator, and metakaolin which is not involved in (or unreacted) geopolymerization at high-temperature. As a result of Rietveld refinement of Si-rich nepheline and low-carnegieite, it was confirmed that two crystalline diffraction patterns were overlapped at 2θ 21° (Figure 5b).

The diffraction pattern of wollastonite (CaSiO_3_, PDF #00-027-0088) was consistent with a newly formed phase in Ca4, Ca8, and Ca16 geopolymers (Figure 6). It is known as the high-temperature crystalline phase that is formed when C-S-H(I) gel is heated at 800 °C and its main diffraction pattern of wollastonite is observed at 2θ 30° [47]. The low reflection of wollastonite was confirmed in Ca4 geopolymer which did not show the C-S-H reflection at room temperature XRD analysis (Figure 6b). Considering these aspects, C-S-H gel was formed in all geopolymers containing calcium hydroxide, though the amount of precipitated C-S-H gel was small and the asymmetric amorphous hump of geopolymer concealed its reflection.

## 4. Conclusions

The effect of calcium hydroxide on the setting behavior and compressive strength of metakaolin-based geopolymers was investigated. The ratio of metakaolin, fumed silica, NaOH, and distilled water in the geopolymers was kept in the formulations (Na_2_O:Al_2_O_3_:SiO_2_:H_2_O = 1:1:4:10) in order to confirm the effect of calcium. The setting of geopolymers was accelerated in proportion to the dosages of calcium hydroxide in up to 4% of the total mix weight. The addition of 2% calcium hydroxide to the mixture was beneficial for the improvement of compressive strength of the geopolymers. The fast setting and enhancement of compressive strength is presumably caused by the fast dissolution of metakaolin and precipitation of C-S-H gel. In the XRD analysis, the cuspidal diffraction peak of C-S-H gel was observed in geopolymers containing 8% and 16% calcium hydroxide. The C-S-H gel is rapidly precipitated at the early stage of reaction and some of the calcium hydroxide remains intact due to the reduction of solubility and common ion effect. The ex-situ high-temperature XRD analysis and Rietveld refinement results showed that geopolymer with a Si/Al ratio of 2.0 and C-S-H gel transformed into Si-rich nepheline and wollastonite, respectively.

## Figures and Tables

**Table 1:** Mix formulation of metakaolin-based geopolymers containing calcium hydroxide. Calcium hydroxide was added from 0% to 16% of the total mix weight.

Sample Name	Chemical Composition	Dosages of Calcium Hydroxide
Ca0	Na_2_O:Al_2_O_3_:SiO_2_:H_2_O = 1:1:4:10	0%
Ca2		2%
Ca4		4%
Ca8		8%
Ca16		16%

**Table 2:** The bulk chemical composition of metakaolin in this study. XRD analysis proved that the TiO_2_ content originated from anatase.

Oxide	SiO_2_	Al_2_O_3_	Fe_2_O_3_	CaO	MgO	K_2_O	Na_2_O	TiO_2_	MnO	P_2_O_5_	Others
wt%	52.64	43.84	0.26	0.03	0.09	0.16	0.35	1.49	0.01	0.08	0.93

**Table 3:** The 7-d compressive strength of metakaolin-based geopolymer. The addition of 2% calcium hydroxide improved the mechanical property of Ca2.

Sample Name	7-d Compressive Strength (MPa)
Ca0	68 (±4.58)
Ca2	76 (±7.21)

## Data Availability

Not applicable.
